# Characterization of the different oligomeric states of the DAN family antagonists SOSTDC1 and SOST

**DOI:** 10.1042/BCJ20200552

**Published:** 2020-09-04

**Authors:** Gregory R. Gipson, Chandramohan Kattamuri, Magdalena Czepnik, Thomas B. Thompson

**Affiliations:** Department of Molecular Genetics, Biochemistry, and Microbiology, University of Cincinnati, Medical Sciences Building, Cincinnati, OH 45267, U.S.A

**Keywords:** BMP, oligomerization, SOST, SOSTDC1

## Abstract

The DAN (differential screening-selected gene aberrative in neuroblastoma) family are a group of secreted extracellular proteins which typically bind to and antagonize BMP (bone morphogenetic protein) ligands. Previous studies have revealed discrepancies between the oligomerization state of certain DAN family members, with SOST (a poor antagonist of BMP signaling) forming a monomer while Grem1, Grem2, and NBL1 (more potent BMP antagonists) form non-disulfide linked dimers. The protein SOSTDC1 (Sclerostin domain containing protein 1) is sequentially similar to SOST, but has been shown to be a better BMP inhibitor. In order to determine the oligomerization state of SOSTDC1 and determine what effect dimerization might have on the mechanism of DAN family antagonism of BMP signaling, we isolated the SOSTDC1 protein and, using a battery of biophysical, biochemical, and structural techniques, showed that SOSTDC1 forms a highly stable non-covalent dimer. Additionally, this SOSTDC1 dimer was shown, using an *in vitro* cell based assay system, to be an inhibitor of multiple BMP signaling growth factors, including GDF5, while monomeric SOST was a very poor antagonist. These results demonstrate that SOSTDC1 is distinct from paralogue SOST in terms of both oligomerization and strength of BMP inhibition.

## Introduction

Bone morphogenetic proteins (BMPs) are the largest subclass of the transforming growth factor-β (TGF-β) family of extracellular signaling proteins, containing ∼15 of the 33 family members identified in mammals [1–4]. BMP proteins regulate a host of fundamental regulatory pathways that are important for bone and organ development, wound healing, and tissue homeostasis [4–10]. BMP signaling is primarily mediated though binding to a heterotetrameric complex of 2 type I and 2 type II membrane-bound serine/threonine kinase receptors (BMPRs) [4,11–14]. Upon ligand binding, the intracellular kinase domain of the type II receptors phosphorylates the type I receptors, triggering intracellular Smad signal transduction cascades [4,15–18]. Phosphorylation of the BMP-specific intracellular Smads (Smad-1, -5, and -9) allows them to bind the partnering co-Smad, Smad-4 [4,15–17,19–21]. This Smad complex associates with other DNA-binding proteins and accumulates in the nucleus to induce transcriptional activation of target genes [4,15–18,21–23]. Given the wide array of different biological functions these proteins govern, it has become apparent that the fine-tuning the activity of these potent signaling molecules is a key component of their biology. Accordingly, BMP signaling is carefully regulated at both the intracellular and extracellular levels. Over the last 25 years, an increasing number of secreted protein antagonists of BMP signaling have been described [24,25]. While differing greatly in terms of structure, these antagonists generally function by directly binding to BMP proteins blocking the receptor binding sites, to prevent the formation of the signaling complexes. In vertebrates, the most common BMP antagonists are noggin, chordin, follistatin, and members of the DAN family [26–31].

**Figure 1.**
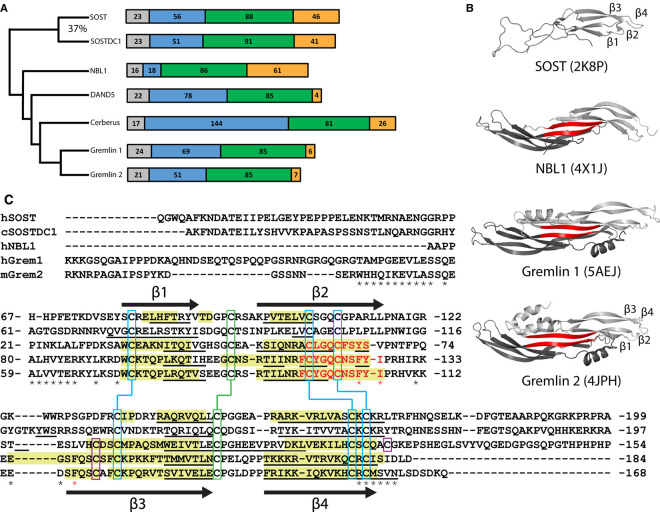
SOSTDC1 in the context of the DAN family. (**A**) A phylogenetic representation of the DAN family. Signal sequence in grey, N-terminal region in blue, cysteine-rich domain (CRD) in green, C-terminal region in orange. SOSTDC1 is most similar to SOST, with 37% identity. (**B**) Currently available solved structures of DAN family members [32,40–42]. Gremlin 1, Gremlin 2 and NBL1 form non-disulfide linked dimers stabilized by extensive hydrogen bonding between antiparallel β-strands (shown in red). (**C**) Multiple sequence structural alignment of the CRDs of the DAN-family, generated using the TCoffee Expresso alignment website and modified to reduce gaps [43]. β-strand regions of solved structures as determined by PyMOL highlighted in yellow. Regions involved in dimer-stabilizing hydrogen bonding in red. Predicted β-strand formation (as determined by SABLE) for all family members underlined [44–46]. Black arrows denoting β-strand based off of the structure of Gremlin 2 (PDB ID:4JPH) [32,40]. Conserved cysteines highlighted in blue, connected to form the cystine-knot. Residues that form the binding interface between Grem2 and GDF5 marked with asterisks, most important residues for robust GDF5 inhibition marked with a red asterisk [47].

The DAN family (Figure 1A) consists of seven members (SOST, SOSTDC1, NBL1, DAND5, Cerberus, Gremlin1 [Grem1] and Gremlin2 [Grem2]) that maintain a conserved Cysteine-Rich Domain (CRD) [29]. The CRD forms a cystine knot that stabilizes the secondary structures of DAN family proteins (Figure 1C) [29]. A number of family members have been shown to inhibit BMP signaling through direct antagonism of the ligand. For example, Grem2 inhibits the BMP ligands, BMP2, BMP4 and BMP7 with IC_50_ values in the low nanomolar range [32,33]. However, DAN family proteins can have activities outside BMP signaling and have been shown to impact other pathways, such as Wnt, VEGF, and even other members of the TGFβ family [34–37]. Specifically, both SOST and SOSTDC1, which share 37% identity, have been shown to inhibit Wnt signaling through interaction with the Wnt co-receptor, LRP5/6 [34,37–39].

SOSTDC1 (historically referred to as Wise, Ectodin, and uterine sensitization-associated gene 1 [USAG1]) was independently discovered through genome wise association studies and functional screens by two groups in 2003 [30,48]. The first of these studies initially categorized SOSTDC1 as a conditional activator or inhibitor of canonical Wnt/β-catenin signaling [30]. The second study identified SOSTDC1 as a BMP antagonist using biochemical techniques [48]. Specifically, direct binding interactions between SOSTDC1 and BMP2, 4, 6 and 7 was measured in the nanomolar range by surface plasmon resonance [48]. Additionally, cell-based alkaline phosphatase assays were used to measure BMP inhibition in an *in vitro* context, indicating robust inhibition of BMP2, 4 and 7 [48]. Subsequent studies have also reported that both SOSTDC1 and SOST inhibit Wnt signaling by competitive binding to the Wnt co-receptor, LRP5/6, corroborating the original findings and demonstrating the importance of SOSTDC1 in regulating multiple different signaling pathways [38,49,50].

In the last decade, DAN family members have been structurally characterized by both NMR and X-ray crystallography. The first NMR structures of SOST revealed a single monomeric protein with a growth factor-like fold of reciprocating β-stands (Figure 1B) [40,51]. Subsequent structural studies on other DAN family members, including Grem1, Grem2 and NBL1, revealed a similar growth-factor like fold, however these proteins were shown to adopt a dimeric form (Figure 1B) [32,41,42]. Interestingly, dimers of Grem1, Grem2 and NBL1 are highly stable and formed through extensive hydrogen bonding along antiparallel β-strands of adjacent chains, specifically the conserved β2 strand (Figure 1B,C) [32,41,42,52]. Although Grem1 and Grem2 contain an odd number of cysteines, it was shown that dimerization is not mediated by a disulfide bond as observed with TGFβ ligands. In fact, NBL1 has an even number of cysteines, shown to form 5 intramolecular disulfide bonds, and still forms a stable dimer. Likewise, SOST and SOSTDC1 have an even number of cysteines. Furthermore, this dimerization appears to play a key mechanistic role in how the DAN family proteins interact with BMP ligands as revealed by the crystal structure of Gremlin2 bound to GDF5 [47]. This study revealed that a single Grem2 dimer can simultaneously bind to two BMP proteins, increasing the avidity of the interaction and potentially introducing an aggregation mechanism that may explain the extreme potency of Grem2 as a BMP inhibitor. Additionally, prior to binding ligand, Grem2 positions a helix over the CRD of the dimer to shield hydrophobic residues that directly contact the ligand (Figure 1C) [47]. Monomeric SOST, in direct contrast, seems have little to no impact on BMP signaling on a biological level, but rather serves exclusively as a functional Wnt antagonist [37].

Taken together, it appears that oligomerization differences which occur within the DAN family might contribute to their functional differences to modulate specific signaling pathways. As previously mentioned, SOSTDC1 is most similar to monomeric SOST, both in terms of sequence identity and biological activity [29]. There is a particularly high degree of similarity between SOST and SOSTDC1 within the CRD, particularly in the region corresponding to the dimer interface and residues associated with BMP antagonism in Grem2 (Figure 1C). In contrast with SOST, a 2009 study by Lintern et al. [50] provided evidence that SOSTDC1 can form a dimer in the presence of chemical cross-linkers. This leads to the question of whether SOSTDC1 exists as a monomer like SOST, or whether it forms a dimer like other DAN family members. As such, determining the oligomerization state of SOSTDC1 is an important component to a complete understanding of the mechanism by which SOSTDC1 inhibits BMP signaling. In this study, we show, using a series of biochemical techniques, that SOSTDC1 forms a highly stable, non-disulfide linked dimer, similar to most DAN family members but distinct from the paralog SOST. In addition, we demonstrate the functional differences between dimeric SOSTDC1 and monomeric SOST, supporting the hypothesis that dimerization is a key component of DAN family specific BMP inhibition.

## Methods

### Recombinant ligands and detection antibodies

BMP7 was purchased from PeproTech. GDF5 was produced in-house using published protocols [47,53]. Primary antibodies used for western blot were polyclonal sheep anti-hUSAG1 (RRID:AB_10972765) and monoclonal mouse anti-hSOST (RRID:AB_2195349) were purchased from R&D. Secondary antibodies used were goat anti-mouse IgG:Alkaline Phosphatase (Calbiochem, RRID:AB_437853) and donkey anti-sheep IgG:Horse Radish Peroxidase (R&D, RRID:AB_562591).

### Generation of SOSTDC1 and SOST expression constructs

The chicken SOSTDC1 (cSOSTDC1) gene (amino acid residues 23–206) was cloned into the pET21a expression vector (pET-cSOSTDC1) using the pCS2-Flag-cSOSTDC1 plasmid as a template (provided by Nobue Itasaki, University of Bristol) [50]. The human SOSTDC1 gene (full-length) was generated by gene synthesis and codon optimized (GenScript), then cloned into pcDNA4 adding a C-terminal tag that included (PreScission Protease (PP) cleavage site (LEVLFQGP), 6× myc-tag and 8× His-tag) to generate the plasmid, pCDNA4-hSOSTDC1-PP-Myc-His. The human SOST/pcDNA3.1+ (full-length) was purchased from Addgene (RRID:Addgene_10842) [54].

### Expression and purification of cSOSTDC1

The pET-cSOSTDC1 vector was transformed into BL21 DE3 (Rosetta). Expression was carried out for 16–18 h at 20°C following induction with 0.5 mM IPTG. Cell pellets contained inclusion bodies of cSOSTDC1 which were resuspended at 4°C in 3 volumes (w/v) of re-suspension buffer (phosphate buffered saline (PBS) supplemented with 0.35 mg/ml lysozyme, 10 µg/ml DNase1 and 2 mM MgCl_2_) and stirred for 30 min at room temperature. The mixture was sonicated and clarified by centrifugation at 7000×***g*** at 4°C for 30 min. The inclusion body pellet was washed twice with PBS supplemented with 0.5% (v/v) Triton X-100 and 1 mM EDTA, and once with PBS. The inclusion bodies were solubilized in 100 mM Tris, 8 M urea, 100 mM Na_2_SO_3_, 10 mM of Na_2_S_4_O_6_, 1 mM EDTA (pH 8.5). cSOSTDC1 was isolated by SEC using a HiPrep Sephacryl S200 16/60 column equilibrated with 0.1 M Tris, 1 M NaCl, 6 M urea, 50 mM MES, 1 mM EDTA (pH 6.0) and subjected to oxidative refolding for 5 days in 100 mM Tris with 150 mM NaCl, 1 mM oxidized glutathione, 1 mM reduced glutathione, 1 mM EDTA and 0.5 M arginine (pH 8.5), similar to Grem2 [33]. Refolded cSOSTDC1 was further purified by heparin affinity chromatography and dialyzed into 20 mM HEPES with 150 mM NaCl (pH 7.5).

### Expression and purification of hSOSTDC1

hSOSTDC1 was transiently expressed for 7 days in Expi293F cells. hSOSTDC1 was purified using heparin resin equilibrated with 20 mM HEPES with 1 mM EDTA (pH 7.5), washed, and eluted with 1 M NaCl. The eluate, containing the hSOSTDC1 protein, was further purified using Ni-NTA XL resin equilibrated with 50 mM NaH2PO4 and 150 mM NaCl (pH 8.0) and, eluted with the addition of 500 mM imidazole. hSOSTDC1 was purified by SEC using a HiLoad Superdex S200 16/60 equilibrated with 20 mM HEPES, 1 M NaCl, 5% (v/v) glycerol (pH 7.5). The myc his tag was removed with PreScission Protease and the untagged protein was isolated by heparin affinity chromatography. Finally, purified hSOSTDC1 was dialyzed into 20 mM HEPES with 150 mM NaCl (pH 7.5).

### Expression and purification of hSOST

The hSOST plasmid was transiently expressed for 4 days in FreeStyle 293F cells. hSOST was purified using SP Sepharose resin equilibrated with 20 mM HEPES (pH 6.5), washed, and eluted with 1 M NaCl, followed by SEC using a Superdex S200 10/300 equilibrated with 20 mM HEPES, 1 M NaCl, 1 mM EDTA (pH 7.5). Purified protein was then dialyzed into 20 mM HEPES, 150 mM NaCl (pH 7.5). hSOST was deglycosylated using DeGlycoMx (QA-Bio) for 4 h at 4°C and subsequently purified by SEC. Purified protein was then dialyzed into 20 mM HEPES,150 mM NaCl (pH 7.5).

### Analytical size-exclusion chromatography (SEC)

Size exclusion chromatography was performed using a Superdex S75 HR 10/300 column at room temperature, flow rate of 0.5 ml/min. The column was pre-equilibrated with 20 mM HEPES,1 M NaCl (pH 7.5) buffer, or 50 mM Citrate and 1 M NaCl (pH 6.5, 5.5, 4.5, or 3.0). Approximately 100–150 µg of protein was loaded for each run. The molecular mass standards used for comparison were bovine serum albumin (BSA) (67 kDa), ovalbumin (43 kDa) and chymo-trypsinogen (25 kDa).

### Analytical ultra-centrifugation (AUC)

Protein samples were dialyzed into AUC buffer (20 mM HEPES, 150 mM NaCl [pH 7.5] or 50 mM Citrate, 150 mM NaCl, [pH 6.5, 5.5, 4.5, or 3.0]) prior to analysis. Sedimentation velocity ultracentrifugation experiments (AUC) were carried out using a Beckman Optima XL-I analytical ultracentrifuge (Beckman Coulter, Fullerton, CA), An60-Ti rotor, and absorbance optics. Samples were loaded into Beckman AUC sample cells with 12-mm optical path two-channel centerpieces, with matched buffer in the reference sector. Absorbance was monitored at peak wavelengths determined using a Nanodrop One spectrophotometer (Thermo). The centrifugation was performed at 48,000 rpm at 20°C, and 300–400 scans were collected at 2 min intervals over 16 h. The observed sedimentation boundaries were fitted to yield a c(s) plot according to the Lamm equation using SEDFIT [55]. Buffer densities, and protein viscosities and partial specific volumes were calculated using SEDNTERP [56]. Molecular mass estimates were determined after determining the coefficient of friction (c(S)) and then fitting the frictional ratio, and are based on a continuous c(M) analysis in SEDFIT.

### Chemical cross-linking

An amount of 4 µg samples of purified protein were incubated with 0.01% (v/v) glutaraldehyde for 20 min at room temperature to induce chemical cross-linking. Native cross-linking reactions were performed in buffer alone (20 mM Hepes,150 mM NaCl pH 7.5]) or buffer with the addition of SDS as indicated. The cross-linking reaction was then neutralized with 1 M Tris (pH 8.0) to a final concentration of 200 mM. Samples were normalized with the highest percentage of SDS prior to PAGE analysis. All conditions were separated by SDS–PAGE under non-reducing conditions.

### Luciferase reporter assay

A BMP-responsive luciferase reporter osteoblast cell line (BRITER, RRID:CVCL_0P40), provided by Amitabha Bandyopadhyay (Indian Institute of Technology), was used to measure BMP activity as previously reported [32,57]. Briefly, cells were grown overnight in α-minimal essential medium with 10% (v/v) FBS, 100 µg/ml hygromycin B, 100 units/ml penicillin, and 100 µg/ml streptomycin in 96 well plate at 37°C in 5% CO2. The medium was replaced with DMEM/high glucose with penicillin, streptomycin, and 0.1% (w/v) bovine serum albumin and starved for a further 4–5 h. The cells were then treated with either exogenous BMP2 (at a final concentration of 1 nM), BMP7 (4 nM) or GDF5 (5 nM) alone or mixed with serial dilution of cSOSTDC1, hSOSTDC1 or hSOST. After 3 h, cells were lysed in 25 µl/well of Passive Lysis Buffer (Promega), mixed with 40 µl/well of luciferase substrate (Promega) and luminescence was measured using a BioTek Synergy H1 plate reader. Data was analyzed using GraphPad Prism, to determine variable slope inhibition curves. IC_50_ values were calculated by fitting data to a nonlinear regression using a least-squares fit, with unconstrained Hillslope, maximum and minimum parameters. All experiments were performed in duplicate.

### In-line SEC-SAXS

SAXS data of cSOSTDC1 were collected using SIBYLS mail-in SAXS service as previously described [58–62]. In brief, cSOSTDC1 protein (at concentrations of 3.4 and 5.6 mg/ml) were mailed to the SIBYLS beamline and isolated over a Shodex 803 SEC column to ensure a disaggregated sample immediately followed by SAXS measurements. ScÅtter (SIBYLS) and the ATSAS program suite (EMBL) were used for data analysis. Comparison of the experimental scattering profiles to known homology models produced from crystal structures (using SWISS-MODEL, with additional unstructured residues modeled) was performed using the FoXS webserver [63–65].

## Results

### Computational analysis of SOSTDC1

To determine if we could gain insight into whether SOSTDC1 would form a monomer or a dimer, we first performed a structural sequence alignment of DAN family members whose structures have been determined using the TCoffee Expression multi-sequence alignment server (Figure 1B,C) [43]. Structures of Grem1, Grem2, and NBL1 have previously revealed a highly stable dimer which included an antiparallel β-strand at the dimer interface. Given this structural feature, we wanted to determine if secondary structure prediction (using the SABLE protein prediction server) across the DAN family when compared with the experimentally derived structures could yield insight into the structure of SOSTDC1 and the potential for a similar dimerization mechanism (Figure 1C) [44–46]. While in general the secondary structure predictions accurately identified all four of the major β-strands, the length of these β-strands were much longer in the experimentally derived crystallographic and NMR structures than those predicted by SABLE. This was particularly apparent for β-strand 2 (β2) where only the N-terminal half of the strand was predicted correctly, leaving out residues in the second half of the intermolecular antiparallel β-strand that stabilizes the dimer (Figure 1C). However, in SOST the secondary structure prediction matches the secondary structure determined by NMR, where the truncated β2 only consists of the a few N-terminal residues, as compared with the dimeric family members. Interestingly, secondary structure prediction of SOSTDC1 suggests even less in the way of β-strand-like features in the dimerization β-strand, β2. This analysis implies that SOSTDC1, similar to SOST, would likely be monomeric. Thus, to reconcile the cross-linking data that SOSTDC1 exists as a dimer with the computational evidence that SOSTDC1 lacks the molecular features of other DAN family dimers required for dimerization, this study characterized the oligomeric state of recombinant SOSTDC1 using a number of orthogonal biophysical approaches and compared these results to purified SOST.

### Production of SOSTDC1 protein

**Figure 2.**
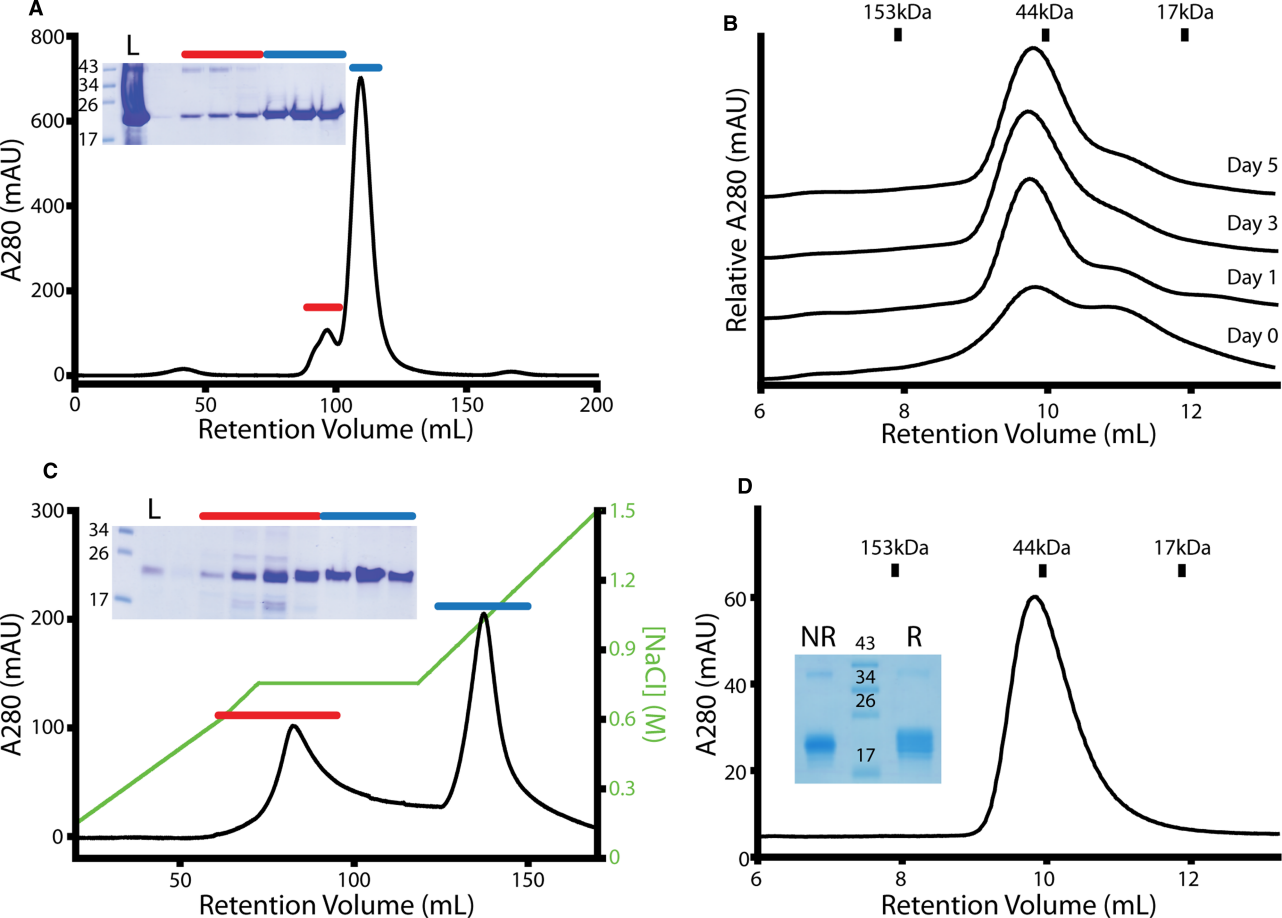
Production of cSOSTDC1. (**A**) Solubilized inclusion bodies purified by size-exclusion chromatography (SEC), and visualized with SDS–PAGE under reducing conditions. Fractions labeled with a blue line were pooled for refolding. (**B**) Transition from unfolded to refolded cSOSTDC1 over the 5-day refolding process, visualized by analytical SEC. (**C**) Refolded cSOSTDC1 purified by heparin affinity chromatography, eluted with a step gradient of increasing NaCl (marked in green), and visualized by SDS–PAGE under non-reducing conditions. Fractions labeled with a blue line were pooled for analysis. (**D**) Pure cSOSTDC1 was evaluated by analytical SEC The protein eluted at a volume consistent with a 43 kDa dimer instead of the 21 kDa monomer shown by SDS–PAGE in both reducing (R) and non-reducing (NR) conditions. Gel ladder values in kDa. Load run in lane L. SEC standards shown for (**B**) and (**D**).

To produce the milligram quantities of SOSTDC1 required for this study, chicken SOSTDC1 (cSOSTDC1) (20.7 kDa) was expressed in *E. coli*. Analysis of expression showed that cSOSTDC1 was exclusively located in the insoluble fraction as inclusion bodies. The inclusion bodies were resolubilized and partially purified by size exclusion chromatography (SEC) (Figure 2A). Since all DAN family members contain a number of cysteine residues that form multiple disulfide binds, cSOSTDC1 was subsequently subjected to oxidative refolding for five days using a protocol similar to previous reports [33]. During this process, daily samples of cSOSTDC1 were analyzed by SEC. Initially, cSOSTDC1 eluted as two distinct peaks, consistent with predicted monomer (21 kDa) and dimer (43 kDa) species (Figure 2B). Over time, the monomer-sized peak slowly decreased and the dimer-sized peak increased. After 5 days, cSOSTDC1 was further purified by heparin affinity chromatography to remove residual misfolded material (Figure 2C). The resulting correctly folded protein sample eluted as a single peak when analyzed by SEC with a molecular mass consistent with a dimer (43 kDa) (Figure 2D). Analysis by SDS–PAGE under non-reducing and reducing conditions shows that the dimer is not covalently linked through a disulfide bond (Figure 2D). There is a trace contaminating band at ∼40 kDa that is present in both reducing and non-reducing conditions. These contaminants are sometimes observed during overproduction in bacteria where the protein is packaged into inclusion bodies, however, since it is less than 3% of the total protein we do not anticipate interference with analysis of the primary species.

### Biochemical and biophysical analysis of SOSTDC1 oligomerization state

**Figure 3.**
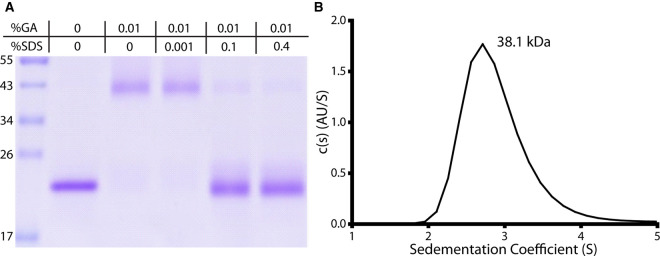
Validation of cSOSTDC1 dimer. (**A**) cSOSTDC1 dimer chemically fixed by glutaraldehyde (GA) cross-linking and visualized by SDS–PAGE in non-reducing conditions. The interaction was disrupted by the addition of SDS. Gel ladder values in kDa. (**B**) Representative sedimentation coefficient distribution profile as determined sedimentation velocity analytical ultracentrifugation. C(s) = 2.89 ± 0.168; C(m) = 38.1 ± 8.4 kDa.

While this analysis indicated that cSOSTDC1 was dimeric, deriving accurate molecular masses from SEC can be confounded by the shape of the proteins and interactions with the column resin. To resolve this issue, we employed orthogonal approaches to confirm the oligomerization state of cSOSTDC1. First, we used the chemical cross-linker glutaraldehyde (GA) to form artificial covalent bonds between amino acid residues in close contact with each other. This served to stabilize any non-disulfide linked oligomerization for visualization by SDS–PAGE. As shown in Figure 3A, glutaraldehyde-treated cSOSTDC1 migrated at ∼43 kDa on a standard SDS–PAGE gel, consistent with the mass of dimeric cSOSTDC1. Furthermore, the dimeric band was disrupted with the addition of sodium dodecyl sulfate (SDS), showing that this interaction is specific and likely dependent on the overall protein fold of cSOSTDC1.

Additionally, we performed sedimentation velocity analytical ultracentrifugation (AUC) to determine, very precisely, the mass of a single particle of cSOSTDC1. Recombinant cSOSTDC1 at a concentration of 500 µg/ml was dialyzed into 20 mM HEPES with 150 mM NaCl (pH7.5), and analyzed by AUC at 48 000 rpm for 300-400 scans (Figure 3B). The normalized continuous sedimentation coefficient distributions c(s), which account for 91% of the observed molecules, from the sedimentation velocity AUC experiments confirmed that cSOSTDC1 was comprised of one single species with sedimentation coefficient of 2.89S and a best-fit frictional ratio of 1.39. These results correspond to a molecular mass of 38.1 ± 8.4 kDa and is consistent with two chains of cSOSTDC1. Experiments were also performed at concentrations of 250 µg/ml and 1 mg/ml to determine if there was a concentration-dependent observation in the molecular mass of cSOSTDC1 (data not shown). All experiments resulted in similar sedimentation profiles, yielding a single sedimentation peak consistent with a dimeric protein configuration.

**Figure 4.**
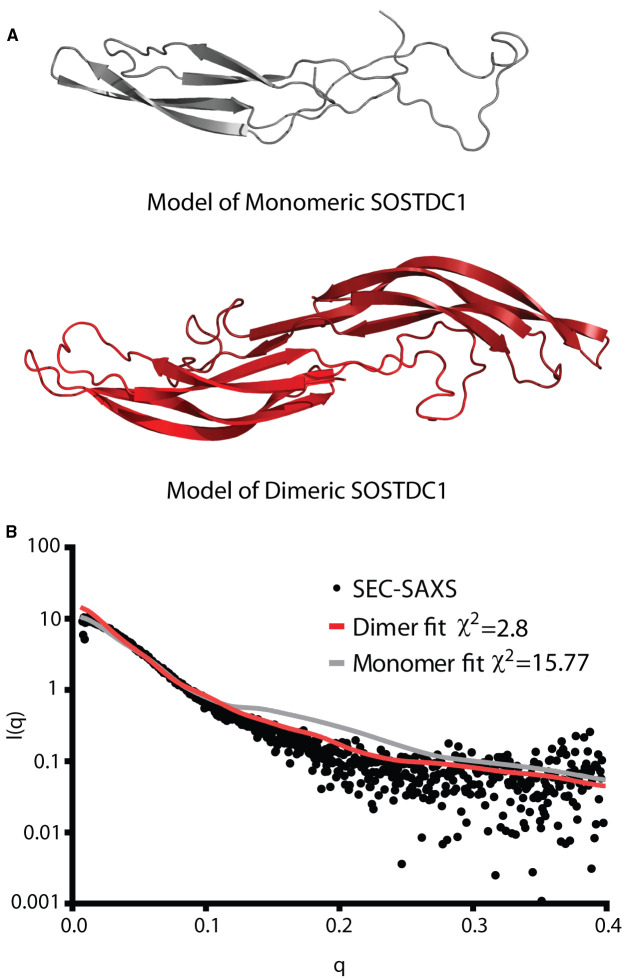
Validation of SOSTDC1 dimer by SEC-SAXS. (**A**) Monomeric and dimeric homology models of SOSTDC1, based off of the structures of SOST (PDB ID:2K8P) and NBL1 (4X1J), generated using SWISS-MODEL [32,40,64]. (**B**) cSOSTDC1 analyzed by in-line SEC-SAXS. The intensity distribution of the SAXS scattering (in black) was compared with theoretical scattering profiles generated using the monomeric and dimeric models of SOSTDC1 using the FoXS server [43,65].

While, there is no experimentally determined high-resolution structure of SOSTDC1 available, low-resolution structural information can be used to further analyze the monomer/dimer structural questions. In conjunction with the SIBLYS beamline at ALS, we performed in-line Size Exclusion Chromatography — Small Angle X-Ray Scattering (SEC-SAXS) on cSOSTDC1. The raw SEC-SAXS data was analyzed using ScÅtter (SIBYLS) and the ATSAS program suite (EMBL), and confirmed to be disaggregated (Supplementary Figure S1). The online FoXS server was used to compare the experimental data to theoretical scattering curves modeled using both monomeric and dimeric homology models of SOSTDC1, based on the structures of SOST (PDB ID: 2K8P), Grem1 (PDB ID: 5AEJ), Grem2 (PDB ID: 4JPH), NBL1 (PDB ID:4X1J), and Norrin (a dimeric protein structurally similar to the DAN-family, that acts as a Wnt signaling agonist, PDB ID:5BQ8) generated using Swiss-MODEL (Figure 4) [32,40–42,63–66]. The monomeric model based on SOST fit the experimental data (χ^2^ = 15.77) substantially worse than any of the dimeric models (NBL1 χ^2^ = 2.82, Grem1 χ^2^ = 3.41, Grem2 χ^2^ = 3.05, Norrin χ^2^ = 3.50). While the SAXS data is not sufficient to distinguish higher resolution structural features, such as whether SOSTDC1 forms a structure substantially more similar to NBL1 than the other dimeric proteins, it clearly supports the hypothesis that SOSTDC1 forms a dimeric as opposed to a monomeric species.

### Analysis of the stability of the SOSTDC1 dimer

**Figure 5.**
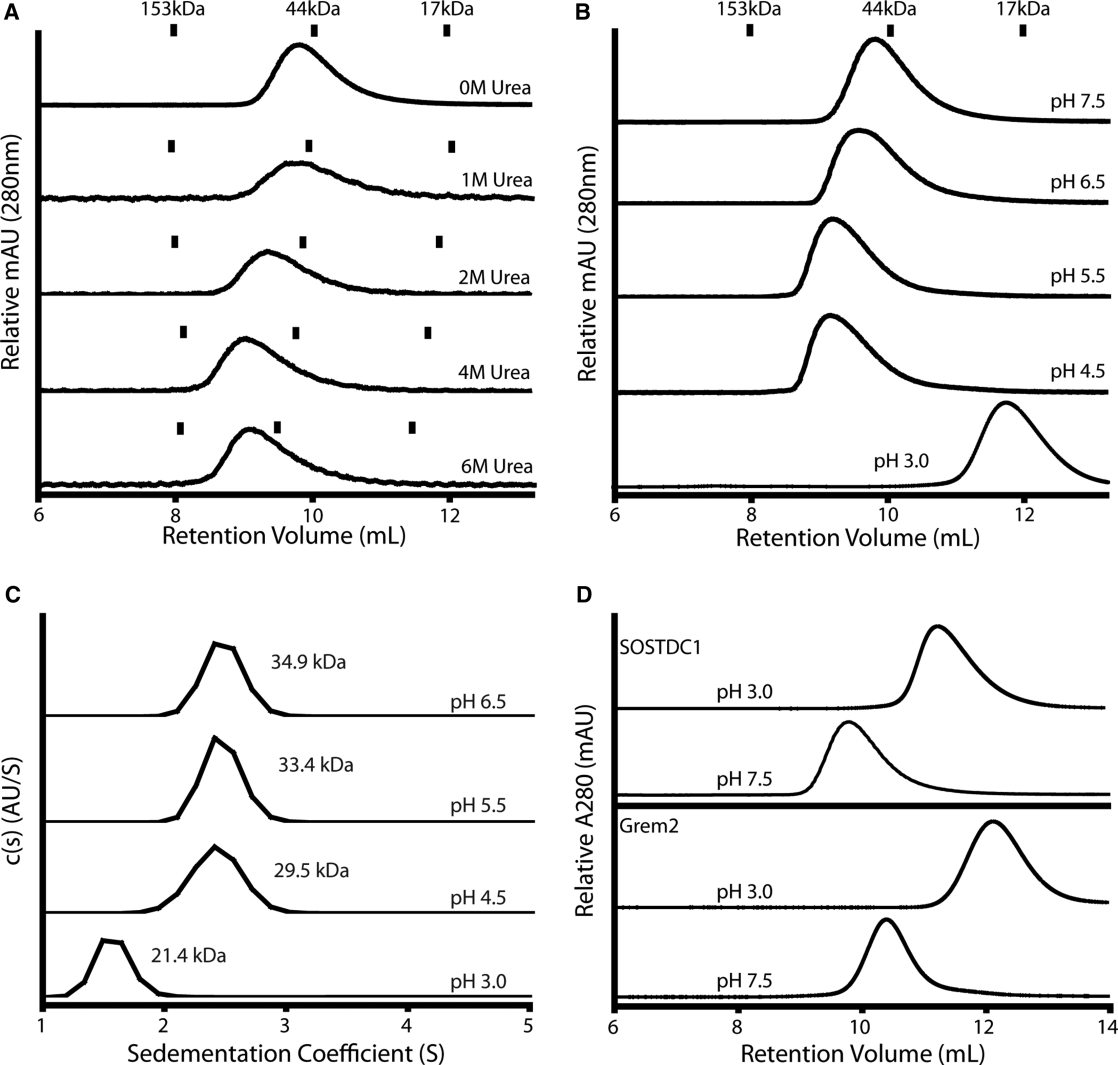
Stability of the cSOSTDC1 dimer. (**A**) cSOSTDC1 dialyzed into increasing concentrations of denaturing urea, analyzed by analytical SEC. cSOSTDC1 maintains its dimeric state even in the presence of 6 M urea. (**B**) cSOSTDC1 dialyzed into buffers of decreasing pH and analyzed by SEC. (**C**) cSOSTDC1 dialyzed into buffers of decreasing pH and analyzed by AUC. (**B** and **C**) SOSTDC1 is dimeric at all pH values expect pH 3.0 where it transitions to a monomer. (**D**) Monomeric cSOSTDC1 and Grem2 at low pH spontaneously reform dimer when returned to pH 7.5, analyzed by SEC. SEC standards are shown for (**A**) and (**B**) as tic marks.

The monomeric nature of SOST, coupled with the secondary structure prediction of cSOSTDC1 that indicated a lower propensity to form the critical intermolecular β2 strand that forms the dimer interface (Figure 1C), compelled us to consider the stability of the cSOSTDC1 dimer. Therefore, we wanted to determine if cSOSTDC1 formed a highly stable, noncovalent dimer as observed for Grem1, Grem2 and NBL1 [32,41,42]. Here, we tested the stability of cSOSTDC1 under several potentially denaturing conditions. Initially, we examined the stability of the cSOSTDC1 dimer in the presence of chemical denaturing reagents. Samples of cSOSTDC1 protein were dialyzed into increasing concentrations of urea, up to 6 M. These samples where then analyzed by SEC, using columns equilibrated with the appropriate urea buffer (Figure 5A). In all of these conditions, the cSOSTDC1 dimer remained remarkably stable, eluting from the column in a single peak with a retention volume consistent with a 43 kDa dimer.

We also determined the stability of the cSOSTDC1 dimer across a range of pH values. Purified cSOSTDC1 was dialyzed into HEPES/NaCl or Citrate/NaCl buffers at different pHs (6.5–3.0) and then analyzed by SEC (Figure 5B). The cSOSTDC1 dimer remained stable down to pH 4.5. However, at pH 3.0 SOSTDC1 eluted as a monomer. Similar observations were shown using sedimentation velocity (Figure 5C). The stability of the SOSTDC1 dimer is consistent with the observed behavior of another DAN family member, Grem2, which has previously been shown to form a highly stable non-disulfide linked dimer, similarly resistant to disruption [52]. Interestingly, when monomeric SOSTDC1 at pH 3.0 was neutralized to a pH of 7.5 and re-evaluated by SEC, the protein eluted as a dimer species with no monomeric species present (Figure 5D). This behavior was also observed for Grem2, indicating that these monomers readily recombine in an ordered manner to form the stable dimer (Figure 5D).

### Biochemical comparison of cSOSTDC1 to natively produced hSOSTDC1 and hSOST

While the bacteria- produced cSOSTDC1 protein allowed us to generate quantities needed for the above experiments, we wanted to confirm our findings using protein that was produced in mammalian cell-culture, and thus retained native folding and glycosylation. A construct containing the gene for human SOSTDC1 (hSOSTDC1) fused to a cleavable C-terminal myc-His tag into HEK Expi293F cells was transiently transfected into Expi293F cells, and the protein was recovered from conditioned medium. The hSOSTDC1, although similar in terms of mature protein sequence (87% sequence identity, 93% similarity) to the bacterial construct of cSOSTDC1, did not have to undergo refolding and contained native glycosylation. As such, it served as an excellent control to validate our bacterially-refolded cSOSTDC1. The human variant of SOSTDC1 was purified to homogeneity from conditioned medium (Supplementary Figure S2A–C) and analyzed by SEC and glutaraldehyde cross-linking. Similar to cSOSTDC1, hSOSTDC1 was also shown to behave predominantly as dimer (Supplementary Figure S2D,E). The protein eluted at a volume consistent with a 60 kDa dimer instead of the 30 kDa monomer shown by SDS–PAGE in both reducing (R) and nonreducing (NR) conditions (Supplementary Figure S2D). The increase in mass of hSOSTDC1 compared with cSOSTDC1 is a product of glycosylation. Additionally, hSOSTDC1 displayed the same pH-dependent dimerization behavior that was observed in cSOSTDC1 and Grem2 (Supplementary Figure S2F).

**Figure 6.**
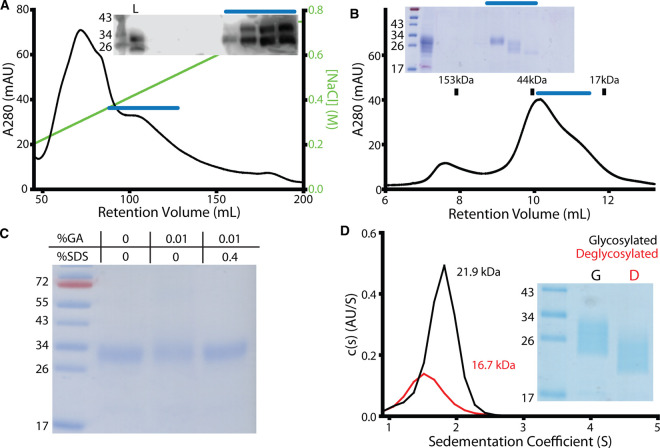
Purification of hSOST and validation of hSOST monomer. (**A**) Human SOST expressed in Expi293F cells, purified by cation exchange chromatography, and visualized by western blot. Fraction marked with blue line was pooled for additional purification. (**B**) Final purification of hSOST by SEC showing a peak corresponding to different glycosylation species (∼30 kDa). The fraction marked with the blue line was pooled for analysis. (**C**) hSOST was incubated with glutaraldehyde (GA), but no higher order complexes were observed by SDS–PAGE under non-reducing conditions. (**D**) Sedimentation velocity AUC of hSOST. Both glycosylated (black, c(S) = 1.77 ± 0.238. c(M) = 21.9 kDa ± 4.4 kDa) and deglycosylated (red, c(S) = 1.55 ± 0.280. c(M) = 16.7 kDa ± 5.4 kDa) sedimented in a manner consistent with monomer. Gel ladder values in kDa. Load run in lane L.SEC standards are shown for (**B**) as tic marks.

The somewhat surprising conclusion that SOSTDC1 formed a highly stable non-covalently linked dimer led us to question whether initial observations showing that, the paralog SOST was monomeric, were correct. To test this, we generated human SOST (hSOST) from media conditioned by transiently transfected FreeStyle 293F cells. This conditioned media was initially purified by Ion Exchange Chromatography (Figure 6A) and then subsequently by SEC (Figure 6B). As with both chicken and human SOSTDC1, the purified hSOST protein was analyzed by glutaraldehyde cross-linking (Figure 6C) and sedimentation velocity AUC (Figure 6D). In all cases, the oligomeric state of hSOST was monomeric, consistent with previous observations [40,51]. These results, using consistent techniques and methodology, show that SOST and SOSTDC1 adopt different oligomeric states, despite similarities in sequence and secondary structure prediction.

### Functional analysis of BMP inhibition

**Figure 7.**
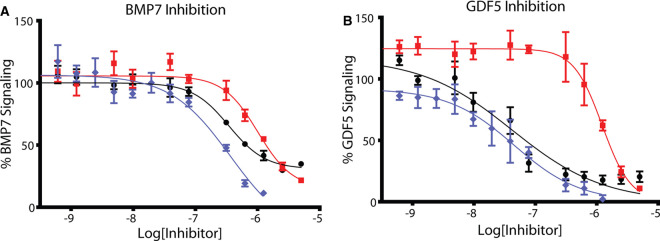
Inhibition of BMP signaling. SOSTDC1 and SOST were measured for BMP inhibition using BRITER osteoblast cells that generate luciferase upon stimulation with BMP ligands. Exogenous BMP7 (**A**), and GDF5 (**B**) was added to the cells after brief incubation with increasing concentrations of DAN family proteins (cSOSTDC1 (black), hSOSTDC1 (blue), and hSOST (red)). All assays were performed in duplicate. IC_50_ values (See Table 1) were calculated by fitting data to a nonlinear regression with a variable slope using a least-squares fit, using GraphPad Prism.

Lastly, we measured the ability of the different DAN family members to inhibit the signaling of BMP ligands using a cell-based luciferase reporter assay. This also served to compare the functional activities of the bacterially produced cSOSTDC1 to the mammalian produced hSOSTDC1. Additionally, these proteins were compared with the activity of monomeric hSOST. We tested these inhibitor proteins against an array of BMP signaling ligands to explore ligand specificity. In brief, serially diluted exogenous inhibitors (cSOSTDC1, hSOSTDC1, and hSOST) were mixed with constant amounts of GDF5 and BMP7 ligands, and added to an osteoblast cell-line (BRITER) stably transfected with the luciferase gene driven by a BMP responsive promoter [57]. Inhibition curves were generated by recording the measured luciferase activity for each concentration of antagonist. As shown in Figure 7 and Table 1, both cSOSTDC1 and hSOSTDC1 inhibited the canonical target BMP7 and GDF5 at sub-micromolar levels with both versions of SOSTDC1 being more potent antagonists of GDF5 signaling. The corresponding IC_50_ values for each titration are shown in Table 1. In contrast, monomeric hSOST was a poor inhibitor of both GDF5 and BMP7 signaling, and only had an impact at very high concentrations of hSOST. Differences between hSOSTDC1 and cSOSTDC1 were minor, with hSOSTDC1 behaving as a slightly more potent inhibitor, likely due to minor species-specific differences between the proteins (91% identity). When compared with previously published data measuring the inhibition of BMP7 and GDF5 by other DAN-family proteins, SOSTDC1 inhibits BMP7 at a rate comparable to NBL1, but inhibits GDF5 even better than both NBL1 and Grem2 (Table 1) [32]. Taken together, these results demonstrate differences in the ability of monomeric hSOST to dimeric hSOSTDC1 and cSOSTDC1 in their ability to antagonize BMP signaling.

**Table 1:** A comparison of DAN family BMP inhibition from current and previous studies, as measured by cell-based luciferase reporter assay

		cSOSTDC1	hSOSTDC1	hSOST	Grem2^a^	NBL1^a^
BMP7	IC_50_	320	300	1500	18	199
	95% conf.	(220–470)	(190–480)	(710–3300)		
GDF5	IC_50_	26	40	1000	92	>10 000
	95% conf.	(18–36)	(27–53)	(750–1400)		

a Values for Grem2 and NBL1 were reported in a previous publication [32] that collected and analyzed data using similar methodology, by measuring the inhibition of luciferase signal in BRITER cells, induced by the addition of exogenous ligand and antagonist proteins. This research was originally published in the Journal of Biological Chemistry. [32]. © the American Society for Biochemistry and Molecular Biology.

IC_50_ values and 95% confidence ranges were calculated by fitting data to a nonlinear regression with a variable slope using a least-squares fit, using GraphPad Prism.

## Discussion

The DAN family consists of seven extracellular proteins, generally categorized as inhibitors of BMP signaling. While certain family members are potent BMP antagonists, such as Grem1 and Grem2, there has been historical discrepancy over the function of SOST and its role in BMP antagonism [37,54]. Follow-up studies identified that SOST was instead a functional inhibitor of Wnt/β-catenin signaling [37]. Thus, certain DAN family members have the ability to inhibit BMP signaling while others appear to inhibit Wnt signaling. Interestingly, SOSTDC1 has been shown to inhibit both BMP signaling and Wnt signaling [34,48]. The ability to impact Wnt signaling has been attributed to a short segment termed the NXI motif that is shared in common with SOST, and has been shown to interact with the Wnt co-receptors LRP5/6 [38]. While NMR structural studies of SOST have described the protein as a monomer, other family members were shown by X-ray crystallography to be dimeric [41,51,52]. It is logical that the dimeric nature of certain DAN family members renders them more potent BMP antagonist due to higher order binding events [47]. Given these differences across the family, we wanted to structurally and functionally characterize SOSTDC1, especially since its nearest DAN family neighbor is monomeric SOST. Thus, this study was initiated to define the molecular state of SOSTDC1 in order to help better understand the mechanism of BMP antagonism in the DAN family.

Through a combination of analytical SEC, chemical cross-linked SDS–PAGE, and sedimentation velocity AUC, we have conclusively demonstrated that SOSTDC1 forms a non-disulfide linked dimer, similar to other DAN-family inhibitor proteins. Additionally, we used these same techniques to further validate previous reports that SOSTDC1 paralogue, SOST, forms a monomer. The dimeric nature of SOSTDC1 in solution was further validated with in line-SEC small angle X-ray scattering. The strongly dimeric behavior of SOSTDC1 is especially interesting when contrasted with its predicted lack of an extended β-strand 2. The secondary structure prediction program SABLE predicts that, much like SOST, SOSTDC1 is likely to have a very short, truncated β2 strand (Figure 1C) that would not provide a scaffold for the numerous hydrogen bonds needed to stabilize the non-covalent dimer. This suggests that either the SOSTDC1 dimer is stabilized in a different manner than other DAN-family members or that the β2 strand of SOSTDC1 is largely stabilized by the dimer interaction itself, producing a longer strand than predicted. Indeed, this latter explanation is suggested by the comparison of SABLE predictions to structurally observed β-strand length for NBL1, Grem1 and Grem2. All three proteins display greater percentages of β-strand in their respective crystal structures than is predicted by SABLE, particularly in the β2 strand that forms the dimer interface (Figure 1B,C). However, the very high degree of similarity between monomeric SOST and dimeric SOSTDC1 at the putative β2 dimer interface suggests that SOSTDC1 might dimerize in a slightly altered manner, which might be somewhat distinct from other DAN-family dimers. It should be noted that a different dimerization interface was observed for the closely related Norrin protein, which is similarly formed through β-strand interactions, but with a unique overall dimer configuration is much more curved with a crescent-like shape [66]. Future atomic-resolution structural studies of SOSTDC1 would be required to investigate the dimerization mechanism of SOSTDC1 with regard to this discrepancy.

We further demonstrated that the dimer formed by SOSTDC1 is highly stable, similar to DAN family members Grem2 and NBL1. Neither the presence of high concentrations of the denaturing reagent urea nor a decrease in pH down to 4.5 were sufficient to disrupt the SOSTDC1 dimer complex, as measured by SEC and AUC. However, the dimer was disrupted when suspended in a pH 3.0 buffer. Interestingly, the SOSTDC1 dimer was shown to spontaneously reform when returned to a neutral pH environment. While it is well established that many proteins will unfold or even denature at very high or low pH, reversible dimer-monomer transition is not commonly observed. Collectively, the high-stability of the SOSTDC1 dimer and the dependence on pH, including the reversible nature of the dimer, indicate a similar dimerization mechanism to other DAN family members that combine through a β-strand ‘zippering’ interaction that forms an extensive network of hydrogen bonds between chains [42].

Different members of the DAN family have shown differential preference for the binding and inhibition of TGFβ ligands. For instance, Grem2 is a potent antagonist of BMP2 and BMP7 with weaker affinity for GDF5 [32,42]. NBL1 antagonizes BMP2 and BMP7 at moderate concentrations, but shows little to no antagonism for GDF5 [32]. Human Cerberus was shown to bind and antagonize BMP6, BMP7, Nodal and Activin B [36]. To provide additional insight into ligand specificity, SOSTDC1 (human and chicken) and SOST were tested for their abilities to inhibit a range of BMP signaling molecules using an *in vitro* luciferase reporter assay system. Both homologous forms of SOSTDC1 inhibited BMP7 (a previously reported target of SOSTDC1) and GDF5 at sub-micromolar concentrations. In contrast, monomeric SOST failed to inhibit either of the tested ligands at sub-micromolar concentrations.

One possible explanation for the differential inhibition of ligands by SOST and SOSTDC1 is that they contain different residues at the putative antagonist-ligand interface. The binding interface and key binding residues of Grem2 for GDF5 were previously determined by the complex structure of Grem2-GDF5 and mutagenesis studies [47]. The N-terminus thread over the ligand into the type I binding pocket, while residues that form the core of interactions at the type II binding interface of GDF5 are located principally between the β2 and β3 strands. Comparing these regions for SOST and SOSTDC shows that the N-terminal residues are not conserved, while resides that bind to the type II interface are conserved, but distinct from those found in Grem2 (Figure 1C). Thus, one possibility to explain the differences in ligand antagonism could come from differences in the N-terminus of SOST and SOSTDC1. However, it is notable that the SOSTDC1 N-terminal tail is also not particularly similar to any of the other DAN-family members, implying that there may not be an obvious binding motif associated with robust BMP antagonism at this site. NBL1, a mediocre inhibitor of BMP7 but better than SOST, almost entirely lacks an N-terminal domain and mutations in this region failed to stop Grem2 from neutralizing BMP [47]. Another explanation could be that dimerization itself is a key component to the mechanism of DAN-family antagonism of BMP signaling by increasing avidity between the antagonists and the ligands. Also, while the contacts of Grem2 with GDF5 are isolated to one chain of Grem2, interactions across the dimer interface might help form the BMP binding epitope. This might be the case for residues in the end of β4, which are pinned between the ligand and adjacent chain of the Grem2 dimer. At this point, whether increased BMP inhibition is through unique contacts, difference in oligomerization, or a combination still needs further investigation.

Functionally, SOSTDC1 has been shown to be important in regulating BMP7 signaling during kidney development and repair [67,68]. Additionally, there is evidence that it serves a similar role in tooth development, although this evidence is muddled by the presence of Wnt5 signaling that SOSTDC1 also antagonizes [69,70]. Interestingly, SOSTDC1 inhibited GDF5 with the strongest potency of all ligands tested, and is a better GDF5 antagonist than all other reported DAN family members, including Grem2 and NBL1 (Table 1). Whether SOSTDC1 blocks GDF5 *in vivo* will have to be explored. Nevertheless, both genes display overlapping expression profiles in the lung and salivary gland, according to the RNA-seq data available from the Human Protein Atlas [71]. Thus, it appears that within the DAN family there exists differential ligand specificity, with SOSTDC1 showing the highest potential to inhibit GDF5 signaling.

In this study we showed that SOSTDC1 forms a highly stable dimer similar to most other DAN family proteins. We also confirmed that SOST forms a monomer and is a relatively poor BMP antagonist, reinforcing its place as a unique member of the DAN family. While both have the ability to inhibit Wnt signaling, SOSTDC1 and SOST have diverged in their ability to antagonize BMP ligands. While it is possible that SOSTDC1 and SOST have altered BMP binding epitopes that render SOSTDC1 a better antagonist, differences in dimerization likely play a critical role in BMP antagonism. Given that most DAN family members are dimeric, it is possible that SOST evolved to be monomeric which reduced its ability to inhibit BMP signaling. Overall, this study provides molecular insight into DAN-family mediated BMP antagonism.

## Abbreviations

AUC: analytical ultracentrifugation
BMP: bone morphogenetic protein
DAN: differential screening selected gene aberrative in neuroblastoma
GDF: growth differentiation factor
SDS–PAGE: sodium dodecyl sulfate polyacrylamide gel electrophoresis
SEC: size exclusion chromatography

## References

[1] Urist M.R. and Strates B.S. (1971) Bone morphogenetic protein. J. Dental Res. 50, 1392–1406 10.1177/00220345710500060601 4943222

[2] Reddi A.H. (2001) Bone morphogenetic proteins: from basic science to clinical applications. J. Bone Joint Surg. Am. 83, S1–S6 10.2106/00004623-200100001-00001 11263660

[3] Wozney J.M. (1992) The bone morphogenetic protein family and osteogenesis. Mol. Reprod. Dev. 32, 160–167 10.1002/mrd.1080320212 1637554

[4] Hinck A.P., Mueller T.D. and Springer T.A. (2016) Structural biology and evolution of the TGF-β family. Cold Spring Harb. Perspect. Biol. 8, a022103 10.1101/cshperspect.a022103 27638177 PMC5131774

[5] Wozney J., Rosen V., Celeste A., Mitsock L., Whitters M., Kriz R. et al. (1988) Novel regulators of bone formation: molecular clones and activities. Science 242, 1528–1534 10.1126/science.3201241 3201241

[6] Hemmati-Brivanlou A. and Thomsen G.H. (1995) Ventral mesodermal patterning in *Xenopus* embryos: expression patterns and activities of BMP-2 and BMP-4. Dev. Genet. 17, 78–89 10.1002/dvg.1020170109 7554498

[7] Tsuji K., Bandyopadhyay A., Harfe B.D., Cox K., Kakar S., Gerstenfeld L. et al. (2006) BMP2 activity, although dispensable for bone formation, is required for the initiation of fracture healing. Nat. Genet. 38, 1424–1429 10.1038/ng1916 17099713

[8] Lowery J. and Caestecker M.D. (2013) BMP signaling and vascular disease. Encyclopedia Biol. Chem. 1, 229–239 10.1016/B978-0-12-378630-2.00356-X

[9] Tomita M., Asada M., Asada N., Nakamura J., Oguchi A., Higashi A.Y. et al. (2013) Bmp7 maintains undifferentiated kidney progenitor population and determines nephron numbers at birth. PLoS ONE 8, e73554 10.1371/journal.pone.0073554 23991197 PMC3753328

[10] Kim H.-S., Neugebauer J., Mcknite A., Tilak A. and Christian J.L. (2019) BMP7 functions predominantly as a heterodimer with BMP2 or BMP4 during mammalian embryogenesis. eLife 8, e48872 10.7554/eLife.48872 31566563 PMC6785266

[11] Yamashita H., Dijke P.T., Huylebroeck D., Sampath T.K., Andries M., Smith J.C. et al. (1995) Osteogenic protein-1 binds to activin type II receptors and induces certain activin-like effects. J. Cell Biol. 130, 217–226 10.1083/jcb.130.1.217 7790373 PMC2120513

[12] Kawabata M. (1998) Signal transduction by bone morphogenetic proteins. Cytokine Growth Fact. Rev. 9, 49–61 10.1016/S1359-6101(97)00036-1 9720756

[13] Heldin C.-H., Moustakas A., Souchelnytskyi S., Itoh S. and Dijke P.T. (2001) Signal transduction mechanisms for members of the TGF-β family. TGF-β and Related Cytokines in Inflammation:11–40

[14] Allendorph G.P., Vale W.W. and Choe S. (2006) Structure of the ternary signaling complex of a TGF-beta superfamily member. Proc. Natl Acad. Sci. U.S.A 103, 7643–7648 10.1073/pnas.0602558103 16672363 PMC1456805

[15] Chaikuad A. and Bullock A.N. (2016) Structural basis of intracellular TGF-β signaling: receptors and smads. Cold Spring Harb. Perspect. Biol. 8, a022111 10.1101/cshperspect.a022111 27549117 PMC5088531

[16] Moustakas A., Souchelnytskyi S. and Heldin C.H. (2001) Smad regulation in TGF-beta signal transduction. J. Cell Sci. 114, 4359–4369 PMID:11792802 10.1242/jcs.114.24.4359

[17] Massagué J. (2012) TGFβ signalling in context. Nat. Rev. Mol. Cell Biol. 13, 616–630 10.1038/nrm3434 22992590 PMC4027049

[18] Abdollah S., Macías-Silva M., Tsukazaki T., Hayashi H., Attisano L. and Wrana J.L. (1997) TβRI phosphorylation of Smad2 on Ser465and Ser467Is required for Smad2-Smad4 complex formation and signaling. J. Biol. Chem. 272, 27678–27685 10.1074/jbc.272.44.27678 9346908

[19] Chen D., Zhao M. and Mundy G.R. (2004) Bone morphogenetic proteins. Growth Fact. 22, 233–241 10.1080/08977190412331279890 15621726

[20] Moustakas A. (2002) From mono- to oligo-Smads: The heart of the matter in TGF-beta signal transduction. Genes Dev. 16, 1867–1871 10.1101/gad.1016802 12154118

[21] Lagna G., Hata A., Hemmati-Brivanlou A. and Massagué J. (1996) Partnership between DPC4 and SMAD proteins in TGF-β signalling pathways. Nature 383, 832–836 10.1038/383832a0 8893010

[22] Pouliot F. and Labrie C. (2002) Role of Smad1 and Smad4 proteins in the induction of p21WAF1,Cip1 during bone morphogenetic protein-induced growth arrest in human breast cancer cells. J. Endocrinol. 172, 187–198 10.1677/joe.0.1720187 11786386

[23] Shi Y., Wang Y.-F., Jayaraman L., Yang H., Massagué J. and Pavletich N.P. (1998) Crystal structure of a smad MH1 domain bound to DNA. Cell 94, 585–594 10.1016/S0092-8674(00)81600-1 9741623

[24] Yanagita M. (2005) BMP antagonists: Their roles in development and involvement in pathophysiology. Cytokine Growth Fact. Rev. 16, 309–317 10.1016/j.cytogfr.2005.02.007 15951218

[25] Mulloy B. and Rider C.C. (2015) The bone morphogenetic proteins and their antagonists. Vitam. Horm. 99, 63–90 10.1016/bs.vh.2015.06.004 26279373

[26] Reem-Kalma Y., Lamb T. and Frank D. (1995) Competition between noggin and bone morphogenetic protein 4 activities may regulate dorsalization during xenopus development. Proc. Natl Acad. Sci. U.S.A 92, 12141–5 10.1073/pnas.92.26.12141 8618860 PMC40312

[27] Sasai Y., Lu B., Steinbeisser H. and Robertis E.M.D. (1995) Regulation of neural induction by the Chd and Bmp-4 antagonistic patterning signals in xenopus. Nature 376, 333–336 10.1038/376333a0 7630399

[28] Hemmati-Brivanlou A., Kelly O.G. and Melton D.A. (1994) Follistatin, an antagonist of activin, is expressed in the Spemann organizer and displays direct neuralizing activity. Cell 77, 283–295 10.1016/0092-8674(94)90320-4 8168135

[29] Nolan K. and Thompson T.B. (2014) The DAN family: modulators of TGF-β signaling and beyond. Protein Sc. 23, 999–1012 10.1002/pro.2485 24810382 PMC4116650

[30] Hsu D.R., Economides A.N., Wang X., Eimon P.M. and Harland R.M. (1998) The Xenopus dorsalizing factor gremlin identifies a novel family of secreted proteins that antagonize BMP activities. Mol. Cell 1, 673–683 10.1016/S1097-2765(00)80067-2 9660951

[31] Ali I.H.A. and Brazil D.P. (2014) Bone morphogenetic proteins and their antagonists: current and emerging clinical uses. Br. J. Pharmacol. 171, 3620–3632 10.1111/bph.12724 24758361 PMC4128061

[32] Nolan K., Kattamuri C., Luedeke D.M., Angerman E.B., Rankin S.A., Stevens M.L. et al. (2015) Structure of neuroblastoma suppressor of tumorigenicity 1 (NBL1). J. Biol. Chem. 290, 4759–4771 10.1074/jbc.M114.628412 25561725 PMC4335214

[33] Kattamuri C., Luedeke D.M. and Thompson T.B. (2012) Expression and purification of recombinant protein related to DAN and cerberus (PRDC). Protein Express. Purif. 82, 389–395 10.1016/j.pep.2012.02.010 PMC3319168 22381466

[34] Itasaki N. (2003) Wise, a context-dependent activator and inhibitor of Wnt signalling. Development 130, 4295–4305 10.1242/dev.00674 12900447

[35] Mitola S., Ravelli C., Moroni E., Salvi V., Leali D., Ballmer-Hofer K. et al. (2010) Gremlin is a novel agonist of the major proangiogenic receptor VEGFR2. Blood 116, 3677–3680 10.1182/blood-2010-06-291930 20660291

[36] Aykul S. and Martinez-Hackert E. (2016) New ligand binding function of human cerberus and role of proteolytic processing in regulating ligand–Receptor interactions and antagonist activity. J. Mol. Biol. 428, 590–602 10.1016/j.jmb.2016.01.011 26802359 PMC7739268

[37] Bezooijen R.L.V., Svensson J.P., Eefting D., Visser A., Horst G.V.D., Karperien M. et al. (2006) Wnt but Not BMP signaling Is involved in the inhibitory action of sclerostin on BMP-Stimulated bone formation. J. Bone Miner. Res. 22, 19–28 10.1359/jbmr.061002 17032150

[38] Bourhis E., Wang W., Tam C., Hwang J., Zhang Y., Spittler D. et al. (2011) Wnt antagonists bind through a short peptide to the first β-Propeller domain of LRP5/6. Structure 19, 1433–1442 10.1016/j.str.2011.07.005 21944579

[39] Guidato S. and Itasaki N. (2007) Wise retained in the endoplasmic reticulum inhibits Wnt signaling by reducing cell surface LRP6. Dev. Biol. 310, 250–263 10.1016/j.ydbio.2007.07.033 17765217

[40] Veverka V., Henry A., Slocombe P., Ventom A., Mulloy B., Muskett F. et al. (2009) Characterisation of the structural features and interactions of sclerostin: molecular insight into a key regulator of Wnt-mediated bone formation. J. Biol. Chem. 284, 10890–10900 10.1074/jbc.M807994200 19208630 PMC2667775

[41] Kišonaitė M., Wang X. and Hyvönen M. (2016) Structure of gremlin-1 and analysis of its interaction with BMP-2. Biochem. J. 473, 1593–1604 10.1042/BCJ20160254 27036124 PMC4888461

[42] Nolan K., Kattamuri C., Luedeke D.M., Deng X., Jagpal A., Zhang F. et al. (2013) Structure of protein related to Dan and Cerberus: Insights into the mechanism of bone morphogenetic protein antagonism. Structure 21, 1417–1429 10.1016/j.str.2013.06.005 23850456 PMC3749838

[43] Armougom F., Moretti S., Poirot O., Audic S., Dumas P., Schaeli B. et al. (2006) Expresso: automatic incorporation of structural information in multiple sequence alignments using 3D-Coffee. Nucleic Acids Res. 34, W604–W608 10.1093/nar/gkl092 16845081 PMC1538866

[44] Adamczak R., Porollo A. and Meller J. (2004) Accurate prediction of solvent accessibility using neural networks-based regression. Proteins Struct. Funct. Bioinformatics 56, 753–767 10.1002/prot.20176 15281128

[45] Adamczak R., Porollo A. and Meller J. (2005) Combining prediction of secondary structure and solvent accessibility in proteins. Proteins Struct. Funct. Bioinformatics 59, 467–475 10.1002/prot.20441 15768403

[46] Wagner M., Adamczak R., Porollo A. and Meller J. (2005) Linear regression models for solvent accessibility prediction in proteins. J. Comput. Biol. 12, 355–369 10.1089/cmb.2005.12.355 15857247

[47] Nolan K., Kattamuri C., Rankin S.A., Read R.J., Zorn A.M. and Thompson T.B. (2016) Structure of gremlin-2 in complex with GDF5 gives insight into DAN-Family-Mediated BMP antagonism. Cell Rep. 16, 2077–2086 10.1016/j.celrep.2016.07.046 27524626 PMC5001929

[48] Laurikkala J., Kassai Y., Pakkasjärvi L., Thesleff I. and Itoh N. (2003) Identification of a secreted BMP antagonist, ectodin, integrating BMP, FGF, and SHH signals from the tooth enamel knot. Dev. Biol. 264, 91–105 10.1016/j.ydbio.2003.08.011 14623234

[49] Li X., Zhang Y., Kang H., Liu W., Liu P., Zhang J. et al. (2005) Sclerostin binds to LRP5/6 and antagonizes canonical Wnt signaling. J. Biol. Chem. 280, 19883–7 10.1074/jbc.M413274200 15778503

[50] Lintern K.B., Guidato S., Rowe A., Saldanha J.W. and Itasaki N. (2009) Characterization of wise protein and Its molecular mechanism to interact with both Wnt and BMP signals. J. Biol. Chem. 284, 23159–23168 10.1074/jbc.M109.025478 19553665 PMC2755721

[51] Weidauer S.E., Schmieder P., Beerbaum M., Schmitz W., Oschkinat H. and Mueller T.D. (2009) NMR structure of the Wnt modulator protein sclerostin. Biochem. Biophys. Res. Commun. 380, 160–165 10.1016/j.bbrc.2009.01.062 19166819

[52] Kattamuri C., Luedeke D.M., Nolan K., Rankin S.A., Greis K.D., Zorn A.M. et al. (2012) Members of the DAN family Are BMP antagonists that form highly stable noncovalent dimers. J. Mol. Biol. 424, 313–327 10.1016/j.jmb.2012.10.003 23063586 PMC3509953

[53] Mueller T.D., Gottermeier M., Sebald W. and Nickel J. (2005) Crystallization and preliminary X-ray diffraction analysis of human growth and differentiation factor 5 (GDF-5). Acta Crystallogr. Sect. F Struct. Biol. Cryst. Commun. 61, 134–136 10.1107/S1744309104031963 PMC1952389 16508114

[54] Semënov M., Tamai K. and He X. (2005) SOST is a ligand for LRP5/LRP6 and a Wnt signaling inhibitor. J. Biol. Chem. 280, 26770–5 10.1074/jbc.M504308200 15908424

[55] Schuck P., Perugini M.A., Gonzales N.R., Howlett G.J. and Schubert D. (2002) Size-distribution analysis of proteins by analytical ultracentrifugation: strategies and application to model systems. Biophys. J. 82, 1096–1111 10.1016/S0006-3495(02)75469-6 11806949 PMC1301916

[56] Laue T.M., Shah B.D., Ridgeway T.M. and Pelletier S.L. (1992) Computer Aided Interpretation of Analytical Sedimentation Data for Proteins In Analytical Ultracentrifugation in Biochemistry and Polymer Science (Harding S. and Rowe A.,eds.), pp. 90–125, Royal Society of Chemistry, Cambridge, UK

[57] Yadav P.S., Prashar P. and Bandyopadhyay A. (2012) BRITER: a BMP responsive osteoblast reporter cell line. PLoS ONE 7, e37134 10.1371/journal.pone.0037134 22611465 PMC3354957

[58] Dyer K.N., Hammel M., Rambo R.P., Tsutakawa S.E., Rodic I., Classen S. et al. (2013) High-throughput SAXS for the characterization of biomolecules in solution: a practical approach. Methods Mol. Biol. Struct. 1091, 245–258 10.1007/978-1-62703-691-7_18 PMC4057279 24203338

[59] Classen S., Hura G.L., Holton J.M., Rambo R.P., Rodic I., Mcguire P.J. et al. (2013) Implementation and performance of SIBYLS: a dual endstation small-angle X-ray scattering and macromolecular crystallography beamline at the advanced light source. J. Appl. Crystallogr. 46, 1–13 10.1107/S0021889812048698 23396808 PMC3547225

[60] Hura G.L., Menon A.L., Hammel M., Rambo R.P., Ii F.L.P., Tsutakawa S.E. et al. (2009) Robust, high-throughput solution structural analyses by small angle X-ray scattering (SAXS). Nat. Methods 6, 606–612 10.1038/nmeth.1353 19620974 PMC3094553

[61] Putnam C.D., Hammel M., Hura G.L. and Tainer J.A. (2007) X-ray solution scattering (SAXS) combined with crystallography and computation: defining accurate macromolecular structures, conformations and assemblies in solution. Q. Rev. Biophys. 40, 191–285 10.1017/S0033583507004635 18078545

[62] Walker R.G., Angerman E.B., Kattamuri C., Lee Y.-S., Lee S.-J. and Thompson T.B. (2015) Alternative binding modes identified for growth and differentiation factor-associated serum protein (GASP) family antagonism of myostatin. J. Biol. Chem. 290, 7506–7516 10.1074/jbc.M114.624130 25657005 PMC4367259

[63] Waterhouse A., Bertoni M., Bienert S., Studer G., Tauriello G., Gumienny R. et al. (2018) SWISS-MODEL: homology modelling of protein structures and complexes. Nucleic Acids Res. 46, W296–W303 10.1093/nar/gky427 29788355 PMC6030848

[64] Schneidman-Duhovny D., Hammel M., Tainer J.A. and Sali A. (2013) Accurate SAXS profile computation and its assessment by contrast variation experiments. Biophys. J. 105, 962–974 10.1016/j.bpj.2013.07.020 23972848 PMC3752106

[65] Schneidman-Duhovny D., Hammel M., Tainer J.A. and Sali A. (2016) FoXS, FoXSDock and MultiFoXS: Single-state and multi-state structural modeling of proteins and their complexes based on SAXS profiles. Nucleic Acids Res. 44, W424–W429 10.1093/nar/gkw389 27151198 PMC4987932

[66] Chang T.-H., Hsieh F.-L., Zebisch M., Harlos K., Elegheert J. and Jones E.Y. (2015) Structure and functional properties of norrin mimic Wnt for signalling with Frizzled4, Lrp5/6, and proteoglycan. eLife 4, e06554 10.7554/eLife.06554 PMC4497409 26158506

[67] Tanaka M., Endo S., Okuda T., Economides A., Valenzuela D., Murphy A. et al. (2008) Expression of BMP-7 and USAG-1 (a BMP antagonist) in kidney development and injury. Kidney Int. 73, 181–191 10.1038/sj.ki.5002626 17943079

[68] Yanagita M. (2005) Uterine sensitization-associated gene-1 (USAG-1), a novel BMP antagonist expressed in the kidney, accelerates tubular injury. J. Clin. Invest. 116, 70–79 10.1172/JCI25445 16341262 PMC1307562

[69] Kiso H., Takahashi K., Saito K., Togo Y., Tsukamoto H., Huang B. et al. (2014) Interactions between BMP-7 and USAG-1 (Uterine sensitization-associated gene-1) regulate supernumerary organ formations. PLoS ONE 9, e96938 10.1371/journal.pone.0096938 24816837 PMC4016158

[70] Saito K., Takahashi K., Asahara M., Kiso H., Togo Y., Tsukamoto H. et al. (2016) Effects of usag-1 and Bmp7 deficiencies on murine tooth morphogenesis. BMC Dev. Biol. 16, 14 10.1186/s12861-016-0117-x 27178071 PMC4866418

[71] Uhlén M., Fagerberg L., Hallström B.M., Lindskog C., Oksvold P., Mardinoglu A. et al. (2015) Proteomics. tissue-based map of the human proteome. Science 347, 1260419 10.1126/science.1260419 25613900

