# Supplementary material for: Characterization of the different oligomeric states of the DAN family antagonists SOSTDC1 and SOST
Source: Biochem J. 2020 Sep 4;477(17):3167–82. doi: 10.1042/BCJ20200552 (PMC7473711; doi:10.1042/BCJ20200552)
Supplement: Supplementary Figures S1-S2 [file BCJ-477-3167-s1.pdf]

Supplementary Figure Legends

Figure S1. SEC-SAXS data quality. cSOSTDC1 inter-atomic pair distribution function P(r), calculated using ScÅtter. P(r) function approach zero smoothly, with D_{max} = 97.0 Å. Analysis of linear Guinier region confirmed non-aggregated sample, with Rg = 31.0 Å.

Figure S2. hSOSTDC1 protein preparation and dimer validation. A) Human SOSTDC1 conditioned medium was purified by heparin affinity chromatography, and visualized by anti-hSOSTDC1 Western blot. The fraction marked with a blue line was then pooled for further purification. A-C) L refers to a sample of the protein that was loaded onto the column. A-E) Gel ladder values in kDa. B) hSOSTDC1 containing an 8x His-tag was further purified by affinity batch preparation using Ni-NTA XL resin (not shown) and then SEC, and visualized by anti-hSOSTDC1 Western blot. The fraction marked with the blue line was pooled for tag removal. C) hSOSTDC1 was incubated with PreScission Protease to remove the tag, and then isolated by heparin affinity chromatography and visualized by anti-hSOSTDC1 Western blot. The fraction marked with the blue line was pooled for analysis. Uncut protein in lane U. D) Pure hSOSTDC1 was analyzed by SEC. The protein eluted at a volume consistent with a 60 kDa dimer instead of the 30 kDa monomer (due to glycosylation) shown by SDS-PAGE in both reducing (R) and nonreducing (NR) conditions. SEC standard shown as tic marks. E) hSOSTDC1 dimer chemically fixed by glutaraldehyde (GA) crosslinking and visualized by SDS-PAGE. This interaction was disrupted by the addition of increasing concentrations of the detergent SDS. F) Monomeric hSOSTDC1 at low pH spontaneously reforms dimer when returned to pH 7.5, analyzed by SEC. Gel ladder values in kDa.

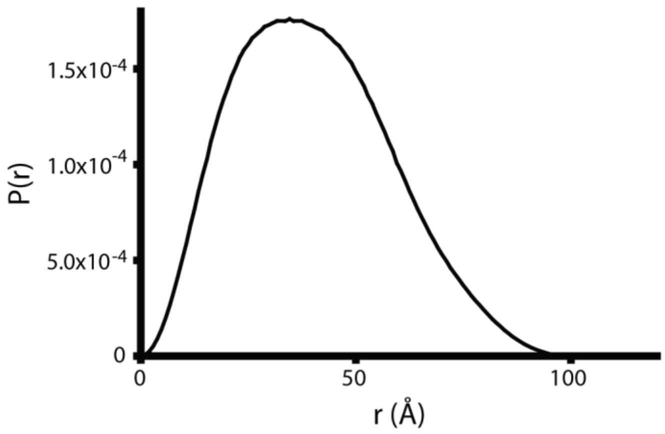
1.5x10-4
1.0x10-4
5.0x10-4
0
P(r)
0
50
100
r (Å)

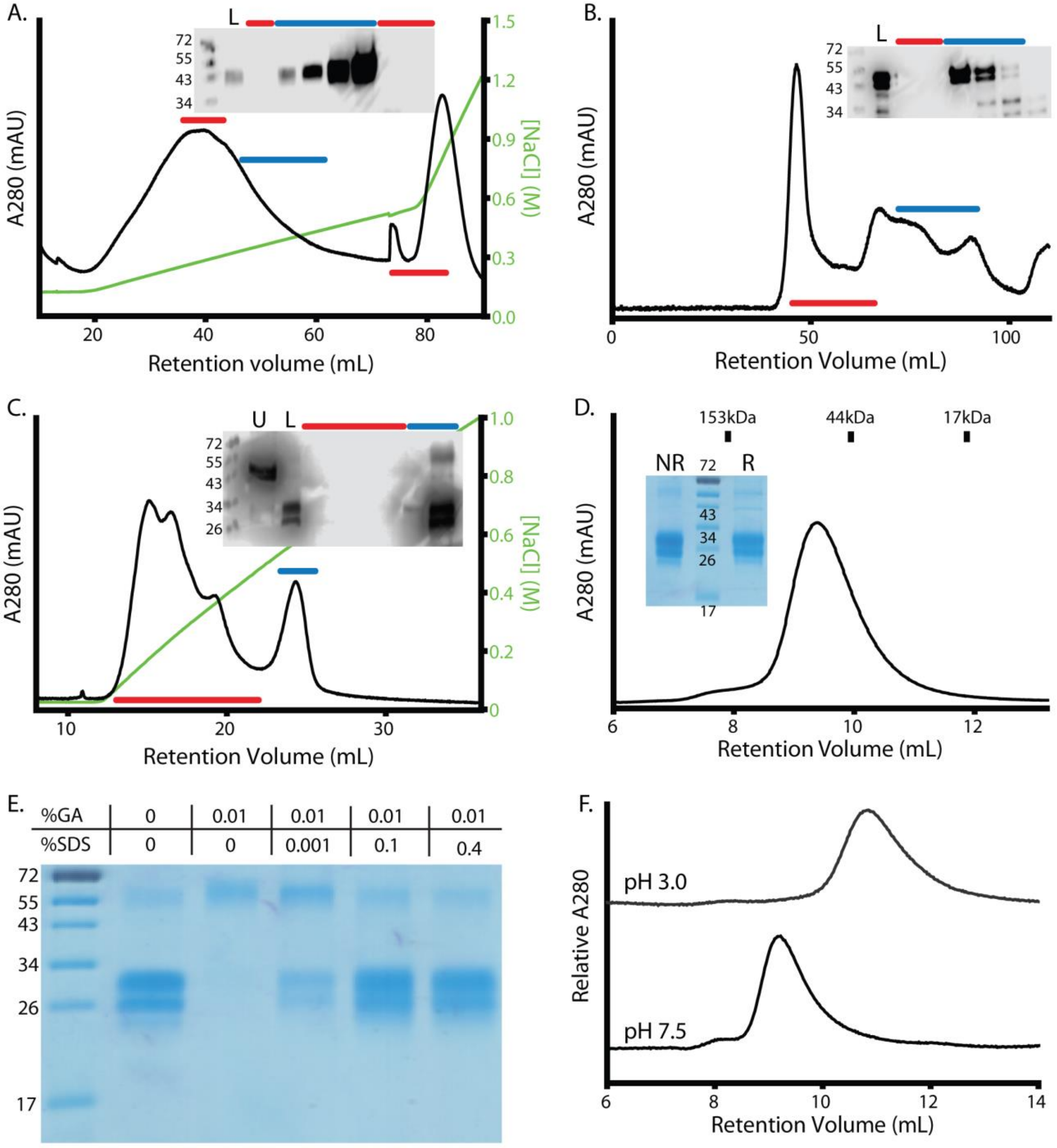

A.
L
72
55
43
34
A280 (mAU)
[NaCl] (M)
1.5
1.2
0.9
0.6
0.3
0.0
20
40
60
80
Retention volume (mL)
B.
L
72
55
43
34
A280 (mAU)
0
50
100
Retention Volume (mL)
C.
U
L
72
55
43
34
26
A280 (mAU)
[NaCl] (M)
1.0
0.8
0.6
0.4
0.2
0
10
20
30
Retention Volume (mL)
D.
153kDa
44kDa
17kDa
NR
R
72
43
34
26
17
A280 (mAU)
6
8
10
12
Retention Volume (mL)
E.
%GA
0
0.01
0.01
0.01
0.01
%SDS
0
0
0.001
0.1
0.4
72
55
43
34
26
17
F.
pH 3.0
pH 7.5
Relative A280
6
8
10
12
14
Retention Volume (mL)
